# The Public Health Implications of the Use and Misuse of Tobacco among the Aboriginals in Canada

**DOI:** 10.5539/gjhs.v5n1p28

**Published:** 2012-10-28

**Authors:** Rotimi Orisatoki

**Affiliations:** 1 School of Public Health, University of Saskatchewan, Saskatoon, Saskatchewan, Canada

**Keywords:** aboriginal, tobacco, misuse, culture, public health, healthcare professionals

## Abstract

Tobacco smoking among the Aboriginal populations is a major public health issue in Canada. It remains a major contributory risk factor to the poor health status as well as years of potential life lost seen among the indigenous people. The use of tobacco has a spiritual importance to the people as a means of making connection to the Creator, but unfortunately tobacco smoking has taken a recreational aspect which has little or no connection with Aboriginal spirituality. The non-traditional use of tobacco is believed by the Elders to be disrespectful to the Aboriginal culture and traditional way of life. There is an increase in rate of use of smokeless tobacco as well as smoking of tobacco among the youth with increase in percentage among females. There are socioeconomic implications as well as adverse health effects of the misuse of tobacco on the Aboriginal people that need to be addressed. The healthcare professionals have a unique role in helping patients to reduce tobacco use within the community through programs that are culturally sensitive and relevant. Successful strategies requires general support from the community and it is very important that some of that support comes from community leaders, including spiritual, professional, administrative and elected policy makers.

## 1. Introduction

Aboriginal peoples in Canada are indigenous inhabitants of Canada, comprising of several groups including First Nations, Métis, and the Inuit. There are over 1.3 million people who self-identify with Aboriginal ancestry, representing approximately 4.4% of the total population (Statistics Canada, 2003).

The rate of tobacco smoking among the Aboriginal people is a major public health issue in Canada (Health Canada, 2002). Tobacco use as a preventable cause of mortality and morbidity remains a major contributory risk factor to the poor health status of the Aboriginal people and years of potential life lost (Wardman & Khan, 2004a). There is an alarming high smoking rates among the Aboriginal communities compared to the general Canadian population according to a study done in 2005, 60 percent of First Nations people smoke compared to 22 percent of the rest Canadians (National Aboriginal Health Organization- First Nations Centre, 2005).

A recently published and reported study done in Canada found a high prevalence of smoking and substance abuse among Aboriginal youth, within ages 14-19 living off-reserve. 24.9 percent of the respondents reported they were current smokers, 2.6 percent were previous smokers, and 72.4 percent were non-smokers. Compared to the non-aboriginal youth, 10.4 percent were current smokers, 1.5 percent former smokers and 88.0 percent non-smokers. Despite this high prevalence of smoking among Aboriginal youth in Canada, there is a paucity of information on their patterns of tobacco use, especially among people living off reserve (Elton-Marshall, Leatherdale & Burkhalter, 2011).

In the traditional Aboriginal societies, tobacco use was primarily for sacred and medicinal purposes until after the European contact that the use of tobacco became highly addictive tobacco. Although the traditional use of tobacco still exists, the misuse of it in form of recreational or habitual use has become a serious health threat to the Aboriginal people.

This paper intends to highlight the public health implications of the misuse of this sacred symbol of importance to the people and the adverse health seen with its misuse. The role of healthcare providers in providing culturally relevant approaches to smoking cessation as well as the various prevention initiatives that includes relevant laws and policies that promote cessation of non-traditional use of tobacco especially among the youth.

## 2. Historical and Cultural Background of Tobacco for the Aboriginal Peoples

The Aboriginal use of tobacco predates the European contact. The use of tobacco had a deep spiritual importance to the people because it is seen as a sacred plant. The traditional use as a means of making a connection to the Creator through prayer and expression of gratitude to the Creator involves the burning of tobacco on a fire or on coals in the form of a hand-made cigar or cigarette to make smoke. In this scenario, the smoke would rise up without the person offering the tobacco inhaling it, thereby making connection with ancestors and with the Creator. Tobacco smoke is also used to cleanse or purify the person participating in a ceremony, or an object or place to be part of that ceremony. The essence of the smoke is to create a spiritual connection between the person offering it and the creator through the spirit receiving it. The Elders or healers also use tobacco in opening prayers in meetings or educational events such as when talking to students on the Aboriginal culture and healing.

Ceremonial pipes are sacred object used in many different rituals and they have symbolic significance to the Aboriginal Peoples. In traditional sense, the most powerful way of communicating with the Spirit world is to smoke tobacco in the sacred pipes. However, because some individuals do see a relationship between a shared pipe ceremony and smoking in the company of others, confusion has developed regarding whether or not smoking has a special place within Aboriginal culture. This is an unfortunate confusion because habitual tobacco use is dangerous to the health of smokers themselves. In contrast, today tobacco smoking has taken a recreational aspect with inhalation usually has little or no connection with Aboriginal spirituality, while the tobacco itself contains numerous harmful chemicals and impurities which are added by cigarette manufacturers (National Native Addictions Partnership Foundation, 2006).

Tobacco may sometimes be placed on the ground as an offering to the earth, or on water, or is left in sacred places. It is not always burned. In the traditional sense, when plants and animals or other objects are taken for use, particularly sacred use, as sign of respect and gratitude by Aboriginal peoples, tobacco is left in its place to acknowledge the gift from Mother Earth. The ceremonial use of tobacco occurred once a day, or in special circumstances two or three times at most. However misuse is seen when tobacco is smoked several times in a day.

The non-traditional use of tobacco is believed to be disrespectful to the Aboriginal traditional and it is considered dangerous and harmful to the health. Although marketing of cigarettes is no longer allowed in Canada, the manufacturers tend to target consumers in form of tobacco products which contains thousands of additives, which has gradually became an increasingly popular, addictive habit that eventually proved to be a primary health hazard among the people (National Native Addictions Partnership Foundation, 2006).

A Saskatchewan First Nations Elder summarised traditional aboriginal worldview of the sacredness, use and misuse of tobacco as follows:

*“Tobacco was seen by our people as a gift from the Creator which would enable us to communicate with him. We were given tobacco because it affected the way we were able to think. It would give us an immediate feeling of heightened awareness because the tobacco we inhaled was that strong. We were given knowledge to fashion a pipe with which we could take very small puffs of tobacco smoke. We would only take small puffs, and then we would immediately blow out the smoke because smoke was not meant to be taken into our body and held there. The smoke needed to leave us in order to rise to the Creator with our prayers and thoughts. If we held it in our body, it would be an unnatural presence there. Immediately after taking the puff of smoke, our minds would race, and our whole body would be affected by this smoke since tobacco is very powerful medicine. It has a specific purpose which must not be abused”* (National Native Addictions Partnership Foundation, 2006).

## 3. The Misuse of Tobacco

The high or growing percentages of the habitual smoking among the Aboriginals are being influenced by lifestyle pressures associated with specific social and economic determinants of health such as low socioeconomic status (Symlie, 2000).

Tobacco misuse can lead to addiction which produces a slight high in the first-time user and it does not impair daily functioning. It is also considered as a legal drug that requires no physician’s prescription. Tobacco smoked in the form of cigarettes is the most popular type of recreational tobacco use. Nicotine is a main component of tobacco and is considered as psycho-active drug in the same category as alcohol and heroin which are considered as hard drugs with additive properties. The psychoactive properties of nicotine modify the brain chemistry producing effects on the mind. Nicotine interacts with specific receptors in the brain tissue, which results in the initiation of metabolic and electrical activity in the brain. The drug also acts on the cardiovascular system and the hormonal system. Nicotine reaches the brain within seven seconds of each drag of a cigarette. Nicotine acts as a stimulant and a depressant, with low doses causes increased in alertness and with high doses tends to relax the user. Nicotine addiction follows the same course as the type of alcohol dependency and other drug habits that graduate to the addictive stage. With repeated use, the body becomes adapted to tobacco and the amount smoked increases, usually until it hits a plateau, such as one pack a day (Retnakaran et al., 2005; Wardman & Khan, 2004b).

Also similar to other addictions, those in the process of quitting smoking experience withdrawal symptoms, both physiological and psychological and the process causes considerable discomfort, agitation and anxiety, making nicotine use to meet the criterion of addiction that indicates that when the user quits, there is a strong chance of starting again, which is referred to as relapse (Enoch, Harris, & Goldman, 2001).

Cigarette dominates both tobacco sales and tobacco smoking. The latest Statistics Canada report showed that the national smoking rate fell from 20.8 percent in 2010 to 19.9 percent in 2011, but the rate in Saskatchewan increased from 22.8 percent in 2010 to 23.8 percent in 2011, with increase seen among the females, while the sales of tobacco in Canada has steady decreased from about 64 billions dollars per year to 31 billion per year (Health Canada, Stat Canada, 2011). The tobacco that is available for commercial sale and use today is not the natural product that was used in traditional ceremonies in the past. Many chemicals are present in commercial tobacco. Some of the chemicals come from fertilizers and from substances that cause the tobacco to burn slowly. There are more than 4000 chemical compounds in the particles of the tobacco smoke, with some of the chemicals causing tobacco-related health problems (Levin, Rezvani, Montoya, Rose, & Swartzwelder, 2003).

Cigarette smoke is a recognised risk factor for development of chronic diseases such as heart diseases, lung disease like emphysema, chronic obstructive pulmonary disease (COPD); diabetes mellitus, oral cancer, laryngeal cancer as well as cancer of the bladder and cervix; emphysema, chronic bronchitis, tooth loss, and gum disease. Smoking may worsens the severity of diabetes complications such as amputation, vision loss, and stroke (National Native Addictions Partnership Foundation, 2006).

A person is exposed to environmental tobacco smoke (often called “second-hand smoke”) in the home when family members or others who share the residence smoke, or when non-smokers must breathe the smoke of others in places where people inevitably congregate, such as the workplace, at schools, at public events etc. Second-hand tobacco smoke can hurt non-smokers as much or more than smokers. This is because there is more than three times the amount of tar, and over six times the amount of nicotine in second-hand smoke than inhaled smoke. Second-hand smoke can therefore affect the future generations as well. If parents or others smoke around a child there is a higher incidence of middle ear problems, coughing, wheezing, and asthma attacks in the children. Infant death and preterm birth has also been associated to maternal smoking (Law et al; 2003; National Native Addictions Partnership Foundation, 2006; Thapar, Fowler, & Rice, 2003).

Smokeless tobacco use includes snuff and chewing tobacco. It may actually be more addictive than cigarette or pipe smoking because the nicotine levels reached in the blood tend to be higher than they are during the smoking of cigarettes. Tobacco chewing incurs a greater hazard of mouth cancer than cigarette smoking. A large number of scientific studies document the cases of mouth, throat and windpipe cancers that are thought to be directly associated with chewing tobacco.. Health problems associated with smokeless tobacco include; bad breath; discoloured teeth; reduced sense of smell and taste; receding gums; wearing of the tooth surfaces; destruction of the supporting bones, making loss of teeth in later life. The use of smokeless tobacco is widespread among the Aboriginal youth, with identified peer pressure and a tendency towards addictive behaviours (National Native Addictions Partnership Foundation, 2006; O’Loughlin, Paradis, & Kim, 2004).

In 1990, Hoover, McDermott, R & Hartsfield found 30 percent of pupils between grades 4 to 12 chewing tobacco or using snuff and 38 percent of them indicate that they either chewed tobacco or used snuff on a regular basis.

## 4. The Current Statistics of the Prevalence Rates

In 2006, Aboriginal Peoples Survey done on the smoking rates in First Nations and Inuit communities showed that 59% of on-reserve First Nations people smoke; 58% of Inuit in the north smoke; almost half of Inuit (46%) who smoke started smoking at age 14 or younger; and the majority of on-reserve First Nations people who smoke (52%) started smoking between the ages of 13 and 16 (Health Canada, 2003).

A more recent study using the Canadian Tobacco Use Monitoring Survey (CTUMS), conducted from February through December 2010, reveals that 17% of the Canadian population aged 15 years and older were current smokers (about 4.7 million Canadian residents). Thirteen percent (13%) reported smoking daily, while 4% reported smoking occasionally. More males (20%) reported smoking than females (14%). Daily smokers smoked an average of 15.1 cigarettes per day (Health Canada, 2010).

## 5. Critical Public Health Implications of the Misuse of Tobacco by Aboriginals

As tobacco smoking remains a public health concerns in Canada, the reduction in the prevalence and incidence rates of smoking will have tremendous benefits on the health status of the indigenous people as well as cost saving on the Canadian health budget (Lemstra et al., 2009).

### 5.1 The Socioeconomic Impact of Smoking

One of the major determinants of cigarette consumption is the cost price of the product. While the bulk of gain for cigarette consumption goes to the manufacturers and sellers of tobacco products, especially the national and multi-national manufacturing and wholesale corporations, much of the burden is carried by the smoker. With alarming frequency, the smoker suffers costs which go far beyond the price of purchasing the thousands of cigarette consumed over time. This correlates with the study done in Massachusetts, USA in which adolescent smoking rate was inversely associated with paternal socioeconomic status (Soteriades & DiFranza, 2003).

Tobacco smoking is much more common among people of lower socioeconomic status, most of which are Aboriginal people in Canada. There are however concerns of cost implications that the increasing prevalence of smoking related diseases, would have on the healthcare budget, as well as the effects of downward social mobility drift of the low income smokers due to their smoking habits (Barbeau, Leavy-Sperounis, & Balbach, 2004).

### 5.2 Nicotine Addiction and Mental Health

Nicotine is a chemical substance in tobacco that causes addiction. Being a highly addictive drug, with a high potent psychoactive substance that induces euphoria, nicotine has both a stimulant and a depressant component. Studies have shown that there is link between tobacco smoking and mental and behavioural problems. A study done among Israeli army observed lower IQ in people who smoked compared to those did not smoke (Weiser et al., 2010). Also prenatal exposure to tobacco smoke has been associated with emotion, impulse and attention problems in later years of the babies (Ghosh, Mishra, Das, Kaushil, & Basu, 2009). The same study linked psychiatric disorders in young adulthood to tobacco usage by mothers during prenatal period.

These studies reinforced the level of concern of the mental health of those who smoke as well as those exposed to smoking among the Aboriginal Canadians.

Effective public health initiatives will involve identifying and targeting the more vulnerable ones, such the youth and pregnant women on the awareness of the risks of tobacco smoking, to understand nicotine addiction, and assist them with smoking cessation programs.

## 6. The Role of the Health Care Professionals

Healthcare professionals play a critical role in reducing tobacco use through prevention programs (US Department of Health and Human Services, 2000). They have a unique opportunity to offer health intervention such as smoking cessation to the population that would help them to stop smoking and its antecedent complications.

Healthcare professionals, by the nature of their position, will be required to take leadership positions in Aboriginal health systems, where mainstream approaches will be combined with more traditional health and healing practices (Smylie, 2000). For Aboriginal healthcare professionals, the likelihood of engagement with Aboriginal patients is increased as compared to a non-Aboriginal health care professional (National Aboriginal Health Organization – First Nations Center, 2003). Even more importantly, Aboriginal healthcare professionals are often seen as role models for their communities. As Aboriginal youth commonly lack such role models in their lives, utilizing Aboriginal healthcare professionals as role models within a smoking prevention program is likely to be an effective and even crucial tool in the reduction of Aboriginal youth smoking rates, though this is a daunting task considering the number of native persons working within the health sector in Saskatchewan. In 2006, the Aboriginal population profile estimated that there were only 1835 Aboriginal healthcare workers that worked among Aboriginal Population of 141,890 (Statistics Canada, 2006). Finally, the development and implementation of effective Aboriginal smoking prevention programs must involve consultation with Aboriginal communities. Aboriginal people must be given the opportunity to participate in interventions in order for health programs to be effective.

The non-aboriginal healthcare professionals can also take similar roles as outlined above, but it may take concerted efforts in form of respect, understanding the culture to gain the trust of the community. They need to demonstrate a good understanding of the peoples’ culture, the role of the Elders as well as the value of their spirituality with tobacco use, which is a critical element in any smoking cessation programs.

## 7. Public Health Implications: Relevant Laws and Policy Issues

There are a variety of prevention programs that have been relatively successful in reducing the percentage of smokers in Canada. Successful strategies requires general support from the community and it is very important that some of that support comes from community leaders, including spiritual, professional, administrative and elected policy makers.

There are several approaches that government can and have taken to reduce tobacco use through prevention policies. Some of the approaches include raising the price of tobacco, complete prohibition on sales of tobacco, stringent legislation and policy on smoke free public places and transport, preventing sales to the minors, treatment of people with addictions, tobacco cessation for pregnant women.

Some of these policies may be difficult to achieve because the history of marginalization the Aboriginal peoples have endured and may prompt an unfavorable response from the First Nations that non-aboriginal governments do not have the right to dictate private behaviours and ordinary community members are unlikely to accept such regulation. Increase in price of tobacco products to tend to reduce consumption but dramatic price hikes do tend to encourage smuggling and an illicit trade in tobacco products. Restricting sales to underage may reduce access to tobacco is been implemented in most countries, but does not necessarily prevent uptake of tobacco use. Interventions aimed at making public places smoke free has shown to be effective in reducing exposure to environmental tobacco smoke especially to children (Ivers, 2008; Law & Tang, 1995; Sandbag, 2006).

Education of the Aboriginal population, especially the youth still remain the most effective policies on tobacco misuse. There is a need for explaining the contrast in tobacco use so prevalent among the youth and helping them to understand that non-ceremonial use of tobacco including inhalation has little or no connection to the Aboriginal culture.

## 8. Smoking Interventions and Strategies Aimed at Reversing High Smoking Rates among Aboriginal People

Aboriginal people tend to take four body approaches to health encompassing the physical, mental, emotional and spiritual. In keeping with the need for a holistic approach, each of the following four areas needs to be addressed to ensure the development and delivery of an effective smoking prevention program for Aboriginal people. It must be culturally appropriate (spiritual), reflect the true role of tobacco (mental), applicable to the youth and provide alternative solution in form of recreation (National Native Addictions Partnership Foundation, 2006).

For Aboriginal people tobacco is a sacred plant used in ceremony and cleansing, which is a spiritual symbol of connection to the Creator. Thus, the negative portrayal of tobacco in mainstream smoking cessation programs creates a great deal of tension. Aboriginal people need a smoking prevention program that is both effective and culturally relevant, such as seen in the Tobacco healing Circle or recovery network where members support one another in a respective and non-judgemental way. The cultural practice of non-interference deems asking for help or offering help as culturally inappropriate. This creates serious barriers to the success of existing smoking prevention programs, most of which utilize group presentations to be effective. This should be done in related substance abuse and addictive behaviour. Another major problem with current mainstream smoking cessation programs is the portrayal of tobacco as negative or evil (McKennitt, 2007).

### 8.1 Understanding the True Role of Tobacco within the Aboriginal Culture

To be effective, Aboriginal people smoking prevention programs must recognize and acknowledge differences between ceremonial tobacco and commercial tobacco use. There is a need for the Elders and Traditional Knowledge Keepers to helping the Aboriginal youth to understand and develop a cultural understanding of tobacco use and the importance of not abusing it (Wardman & Khan, 2004b). The true understanding of the role of tobacco within the Aboriginal culture will assist the youth to connect closely to their culture and seek healing as they connect to their Creator.

In Aboriginal culture, tobacco is not always burned. It may be placed in sacred grounds as offering to the Creator on the water. This knowledge can be used as public health messages when it is being promoted by the Elders as well as healthcare providers. Also the ceremonial use of tobacco occurred once a day or two to three times in special ceremonies, hence as healthcare providers, we can infer that those who smoke more ten or more tobacco per day are abusing the use of tobacco according to the Aboriginal culture.

Some of the Aboriginal youth equate smoking to growing up to live independently like adults. Such idea is propagated in environments where child abuse is rampant. Aboriginal siblings are usually quite close and very influential in each other’s decision-making. The trauma of residential schools has left many modern Aboriginal parents with inadequate parenting skills which may compound the smoking habits. Targeting youth and their parents will help slow down the snowball effect of smoking uptake and nicotine dependence among youth in Aboriginal communities; a problem that is currently rolling out of control. Recreational and physical activities such as swimming, camping, dancing and exercise is an important part of helping Aboriginal youth to remain smoke free (Yakiwchuk, Stasiuk, Wiltshire, & Brothwell, 2005).

## 9. Conclusion

The Public health framework as outlined in the population health model is a strategy for improving the health of a specific nation, group or community, which focuses on the full range of personal and associated factors that influence health and the way those factors work together to contribute to the health and wellbeing of a given population as a whole. This model reflects the wisdom of the Medicine Wheel, employed by Aboriginal culture prior to contact with the Europeans and which is still used by many Elders and Traditional healers as a basis for understanding and giving counsel to people on matters affecting their health, spiritual peace and general wellbeing. In viewing of those adhering to the population health approach, health is understood to be influenced by many factors, which are known as determinants of health or more commonly, health determinants. These determinants of health impact in different ways and in varying degrees on the individual through his or her lifetime (Public Health Agency of Canada, 2001). As health professionals, the onus lies on us to use this framework to address the misuse of tobacco that is gradually becoming a public health epidemic among the Aboriginal people.
